# The protective role of green spaces in mitigating myopia prevalence

**DOI:** 10.3389/fpubh.2024.1473995

**Published:** 2024-10-04

**Authors:** Guy Barnett-Itzhaki, Zohar Barnett-Itzhaki, Daphna Mezad-Koursh

**Affiliations:** ^1^Division of Ophthalmology, Tel Aviv Sourasky Medical Center, Tel Aviv, Israel; ^2^Faculty of Medical and Health Sciences, Tel Aviv University, Tel Aviv, Israel; ^3^Ruppin Research Group in Environmental and Social Sustainability, Ruppin Academic Center, Emek Hefer, Israel

**Keywords:** myopia, green spaces, NDVI, school-level myopia prevalence, epidemiology, refractive error

## Abstract

Near-sightedness, or myopia, is becoming more prevalent worldwide, but its etiopathogenesis is not fully understood. This review examines the potential protective role of green spaces in reducing myopia prevalence among children and adolescents, based on recent epidemiological studies from various countries. The studies consistently used the Normalized Difference Vegetation Index (NDVI) to quantify green space exposure. The analysis reveals a significant inverse relationship between exposure to green space and the risk of developing myopia, across multiple studies. For example, a 0.1 increase in NDVI within various buffer zones around schools was associated with a 6.3–8.7% reduction in myopia prevalence. Higher residential greenness within a 100-meter buffer around homes was linked to a 38% reduction in the risk of developing myopia among preschool children. The protective effect was observed across different age groups, from preschoolers to high school students. Urban planning factors, such as the size, connectivity, and aggregation of green spaces, also influenced myopia risk. These findings suggest that increasing access to green spaces in urban environments may be an effective strategy for myopia prevention, with important implications for public health and urban planning policies.

## Background

Myopia, commonly known as short sightedness, is a type of refractive error, where distant objects appear blurred, while near vision remains relatively unaffected. This condition occurs when the eye grows too long from front to back, causing light rays to focus in front of the retina rather than directly on it. Myopia has become a global public health concern, with its prevalence rising dramatically over the past few decades, particularly in East Asian countries (1).

High myopia can lead to several serious eye health complications, including: Retinal detachment, Glaucoma, Cataracts, Myopic maculopathy, Choroidal neovascularization, Posterior staphyloma, increased risk of open-angle glaucoma, and earlier onset of cataracts (2, 3).

The rising prevalence of myopia, especially among children and adolescents, has prompted researchers to investigate various environmental factors that may contribute to its development and progression. Among these factors, the role of green spaces in urban environments has emerged as a topic of significant interest. Green spaces, defined as open areas with vegetation such as parks, gardens, and natural reserves, are integral components of urban ecosystems, providing numerous health benefits to residents (4, 5). Green spaces encourage outdoor activities, increasing exposure to natural light and providing visual stimulation at various distances, both of which are protective factors against myopia development. Natural light stimulates the release of dopamine in the retina (6), which may help regulate eye growth and prevent excessive elongation of the eyeball, a key factor in myopia development. Additionally, green spaces may promote increased physical activity and reduce time spent on near-work activities, such as using electronic devices (7).

Recent epidemiological studies have investigated on the role of green spaces in mitigating myopia, and have suggested a potential protective effect of green spaces against the development of myopia in children and adolescents. This association is particularly intriguing in light of the concurrent trends of increasing urbanization and decreasing access to natural environments in many parts of the world. Understanding the relationship between green spaces and myopia could have far-reaching implications for urban planning, particularly in the design of areas around schools and kindergartens, as well as for public health policies, and strategies to mitigate the growing myopia epidemic.

The purpose of this concise review is to examine the current evidence linking green spaces to myopia. We aim to synthesize findings from various studies that have addressed this relationship (Table 1), focusing on the methodologies used to quantify exposure to green spaces, the strength and consistency of the observed associations, and finally, discussing the potential mechanisms underlying the potential protective effect of green spaces. Our aim is to inform evidence-based strategies for creating healthier urban environments that support optimal visual development in children and adolescents.

**Table 1:** Integrated results of the association between myopia and green spaces.

Region / country	Year/s of data collection	Number of participants	Age of participants	Findings	Reference
Hyderabad, India	2000–2001	4,074	7–15 years old	Lower rates of myopia in rural areas (4.1%) in comparison to urban areas (7.4%)	Dandona et al. (9)
Barcelona, Spain	2012–2015	2,727	7–10 years old	IQR increase in exposure to green spaces at home and school was associated with 14%/27% reduction in spectacle use, respectively.	Dadvand et al. (10)
Shenzhen, China	2014	53,575	1–3 years old	higher residential greenness within a 100-meter buffer around the home was associated with a 38% reduction in the risk of myopia	Huang et al. (12)
Shenzhen, China	2016–2017	138,735	Grades 1–4	one-unit increase in GSMI led to 9.8% reduction in myopia	Yang et al. (13)
Shanghai, China	2021	1,727,709	3–20 years old	greenness within a 1,000-meter radius of schools significantly reduced the odds of myopia (OR: 0.299)	Wang et al. (14)
Beijing, China	2021	13,380	High school students	A 0.1 increase in NDVI (across 500 m, and 1,000 m buffers) was associated with myopia reductions ranging of 16%/ 12%, respectively.	Zhang et al. (15)
Tianjin, China	2021–2022	1,245,271	Primary schools, junior high schools, and senior high schools (average age of 11.6 years old).	0.1 increase in NDVI within 250, 500, and 1,000 meters of the school was associated with a 6.3, 7.7 and 8.7% reduction in myopia prevalence, respectively.	Lu et al. (16)
Tianjin, China	2021–2023	137,087	Grades 1–6	Each one-unit increase in the urban score was linked to a higher risk of developing myopia over 1 year (OR = 1.09) and 2 years (OR = 1.53)	Li et al. (17)

## Results

### Assessment of green spaces

Many of the studies included in the review utilized the Normalized Difference Vegetation Index (NDVI) for green space quantification. This index estimates vegetation density based on land surface reflectance data captured in visible and near-infrared wavelengths. Its effectiveness for large-scale ecological monitoring of vegetation dynamics is well-established, since its introduction by Rouse et al. (8).

The underlying principle of NDVI methodology leverages the spectral properties of vegetation. Chlorophyll in healthy plants absorbs red light (630–690 nm) within the visible spectrum, while reflecting near-infrared radiation (760–900 nm). This differential reflectance between red and near-infrared wavelengths serves as a proxy for both the quality and intensity of greenness. NDVI is calculated using the following formula:

NDVI = (NIR-Red)/(NIR + Red).

NIR and Red represent spectral reflectance values in the near-infrared and visible red bands, respectively. NDVI values typically range from −1 to 1. Areas with negative values generally correspond to water bodies, while values between 0 and 0.1 reflect barren land (rock, sand, or snow). Values between 0.2 and 0.3 represent shrub and grassland ecosystems, and higher positive values signify denser green vegetation cover.

### Green spaces and myopia

A study on myopia in children living in a rural population in India utilized a cross-sectional design to assess the prevalence of refractive errors and visual impairment in school-aged children (7–15 years old). Data collection included visual acuity measurements, retinoscopy, autorefraction, and subjective refraction. The researchers identified an association between myopia and several factors: female gender, older age, and levels of parental education, specifically the father’s education. The prevalence of myopia differed between the rural population, with myopia rate of 4.1%, and the urban population in New Delhi, with myopia rate of 7.4% (9). This difference was explained by the assumption that rural environments are greener than urban ones, effecting myopia rates in a respective manner.

A study conducted in Barcelona in 2012–2015, evaluated the impact of exposure to green spaces on the use of spectacles among 2,727 schoolchildren aged 7–10 years (10). The authors considered the use of spectacles as a surrogate for myopia, since decreased visual acuity during childhood is commonly associated with the onset of myopia (11). The study measured exposure to green spaces at home or school environment as well as during commuting, using NDVI. Additionally, it obtained data on time spent playing in green spaces using questionnaires. Statistical analyses revealed that an interquartile range (IQR) increase in the exposure to green spaces at home, school, and during commuting, was associated with a 14% (95% CI:2–26%), 27% (95% CI:6–44%), and 20% (95% CI:5–33%) reduction in the use of spectacles, respectively. In longitudinal analyses, these reductions were 23% (95% CI:4–39%) for home and 34% (95% CI:2–55%) for school. Additionally, an IQR increase in the time spent playing in green spaces corresponded to a 28% (95% CI:7–45%) reduction in the likelihood of spectacles use. These findings indicate that higher exposure to green spaces is associated with a decreased likelihood of spectacles use among children, suggesting a potential protective effect against myopia. This association remained robust across various sensitivity analyses, adjusting for socioeconomic status and other potential confounders (10).

A study conducted in 53,575 preschool children (1–3 years old) from Shenzhen, China, assessed the impact of residential greenness on visual health outcomes such as myopia and astigmatism. Data was collected from 2014 with annual follow-ups. The researchers found that higher residential greenness within a 100-meter buffer around the home was associated with a 38% reduction in the risk of myopia (Add adjusted odds ratio (AOR): 0.62, 95% CI:0.38–0.99) and similarly reduced the risk of astigmatism within 100, 250, and 500-meter buffers (AORs: 0.55, 0.59, and 0.61 respectively) (12).

Another study conducted in Shenzhen, utilized high-resolution satellite images from the Gaofen-2 satellite to analyze landscape metrics related to green spaces morphology (13). The baseline data was collected in 2016–2017 and follow-up data was obtained in 2018–2019. The study included 138,735 students in grades 1–4. This study revealed that certain characteristics of green spaces morphology, including larger green space areas, better connectivity between green patches, and more aggregated green spaces, were associated with a reduced risk of myopia among school children. The researches found that a one-unit increase in myopia-related green space morphology index (GSMI) was associated with a 9.8% (95% CI: 4.1–15.1) reduction in the risk of incident myopia (*p* < 0.001). This association persisted after adjusting for outdoor time, screen time, reading time, and parental myopia (AOR, 0.88; 95%CI: 0.80–0.97; *p* < 0.01). Additionally, the analysis showed that a well-designed green space layout, incorporating several specific features, correlated with slower progression in the prevalence of myopia among schoolchildren. These findings collectively suggest that optimizing the morphology of green spaces could potentially reduce the burden of myopia among children, emphasizing the importance of environmental factors in myopia prevention strategies (13).

A study conducted in Shanghai, China in 2021, investigated the relationship between environmental factors and myopia in a large cohort of children and adolescents aged 3–20 (14). The study analyzed visual acuity and non-cycloplegic refraction data from 1,727,709 participants attending 3,399 schools and kindergartens. The research utilized GIS-based data to assess environmental features around these institutions, including green spaces, building density, and the proximity of food outlets. The researchers employed logistic mixed-effect models, adjusting for potential confounders, and performed mediation and sensitivity analyses to ensure robust results. Key findings indicated that greenness within a 1,000-meter radius of schools significantly reduced the odds of myopia (OR: 0.299, 95% CI: 0.249–0.357, *p* < 0.001), with 2.7% of this effect mediated by increased outdoor time. Conversely, higher building density was associated with a significantly increased risk of myopia (OR: 2.764, 95% CI: 2.168–3.525, *p* < 0.001), with 6.2% of this effect linked to reduced outdoor time. The presence of fast-food outlets had only a weak positive association with myopia (OR: 1.001, 95% CI: 1.001–1.001, *p* < 0.001). The effects varied by age and gender, and were more pronounced in the 7–12 year age group. The findings suggest that urban planning and school environments should prioritize green spaces to mitigate the risk of myopia in children (14).

Another study conducted in Beijing, China in 2021 examined the effects of green spaces around schools on individual myopia risk and school-level myopia prevalence (14) among 13,380 high-school participants, using NDVI of images covering 48 school plots within a 2-year period. The average NDVI values within 500 m and 1,000 m buffers around schools were extracted to assess the impact of greenness on myopia risk. The results indicated that a 0.1 increase in the NDVI of 500 m and 1,000 m buffers correlated with a significant reduction in personal myopia risk among high school-aged adolescents. Specifically, the adjusted 500 m buffer NDVI was associated with a 16% decrease in personal myopia risk, while the 1,000 m buffer NDVI demonstrated a 12% reduction. Moreover, the study found that the NDVI of the 500 m buffer around schools significantly was associated with lower school-level myopia prevalence by 15%. Subgroup analyses revealed significant effects in various categories, including schoolgirls, junior high students, individuals of Han nationality, and those with 1- and 3-year exposure times. These findings underscore the importance of green space surrounding schools as a protective factor against myopia in adolescents (15).

A paper based on data collected in Tianjin, China in 2021–2022 investigated the relationship between socioeconomic status (SES), green space exposure, and myopia prevalence among school-aged students. The study included 1,245,271 students from 16 districts in Tianjin, with an average age of 11.6 years. Participants were from primary schools, junior high schools, and senior high schools. Greenness levels around schools were assessed using NDVI based on satellite remote sensing data. Generalized linear mixed effects models were used to analyze the data, adjusting for students’ sex, years of education, and the school’s geographical location. It was found that students living in low SES areas had the highest prevalence of myopia (60.7%) in the most recent screening in 2022, compared to those in intermediate SES areas (22.7%). Furthermore, it was found that a 0.1 increase in NDVI within 250, 500, and 1,000 meters of the school was associated with a 6.3% reduction in myopia prevalence (OR = 0.937; 95% CI: 0.915-0.960), 7.7% (OR: 0.923; 95% CI: 0.900–0.946), and 8.7% (OR: 0.913; 95% CI = 0.889, 0.937), respectively. The findings suggest that increased exposure to green spaces is associated with a lower prevalence of myopia among school-aged students (16).

A study conducted among 137,087 schoolchildren in Tianjin, China, aimed to investigate the relationship between urbanization and myopia. The research involved a cohort of students from grades 1 through 6, and data was collected over a two-year period from March 2021, to April 2023. Vision exams were conducted at three points during the study to assess the incidence, progression, and severity of myopia, which was defined as a spherical equivalent refraction (SER) of −0.50 diopters (D) or less. The researchers developed an urban score using satellite data and exploratory factor analysis based on four environmental variables: population density, night light index, enhanced vegetation index, and walking time to the nearest hospital. The statistical analysis included generalized mixed linear models to evaluate the associations between the urban score and myopia outcomes, adjusting for various factors such as age, sex, grade, and school socioeconomic status. The results indicated a significant positive association between urbanization and myopia incidence. Each one-unit increase in the urban score was linked to a higher risk of developing myopia over 1 year (OR: 1.09; 95% CI: 1.01–1.15; *p* = 0.02) and 2 years (OR: 1.53; 95% CI: 1.50–1.57; *p* < 0.001). Conversely, higher urban scores were associated with reduced myopia progression (OR: 0.84; 95% CI: 0.82–0.86; *p* < 0.001 for 1 year; OR: 0.73; 95% CI: 0.70–0.75; *p* < 0.001 for 2 years). The study highlights the dual role of urban environments in increasing the incidence and decreasing the progression of myopia, suggesting the need for targeted myopia control strategies in different urbanization contexts (17).

## Discussion

The collective findings from the reviewed studies provide compelling evidence for a potential protective effect of green spaces against myopia in children and adolescents. This relationship appears to be consistent across various geographical locations, predominantly in China but also observed in European cities, like Barcelona.

The studies consistently utilized the Normalized Difference Vegetation Index (NDVI) as a primary metric for quantifying green space. This standardized approach allows for comparability across studies and regions. Yang et al. (13) expanded on this by incorporating more detailed landscape metrics, revealing that both the quantity and the quality and arrangement of green spaces play crucial roles in myopia prevention. The findings suggest that larger, more connected, and more aggregated green spaces are more effective in reducing myopia risk. This highlights the importance of careful and informed urban planning in improving public health outcomes.

Several studies demonstrated a dose–response relationship between exposure to green spaces and myopia risk. For instance, Lu et al. (16) found that a 0.1 increase in NDVI within various buffer zones (250 m, 500 m, 1,000 m) around schools was associated with a 6.3%, 7.7% and 8.7%, respectively, reduction in myopia prevalence. This consistent pattern across different buffer sizes strengthens the evidence for a causal relationship between green space exposure and myopia prevention.

Moreover, the protective effect of green spaces appears to be consistent across various age groups, from preschool children (12) to high school adolescents (15). The magnitude of the effect may vary with age. Wang et al. (14) noted that the impact was more pronounced in the 7–12 year age group, suggesting a potentially critical window for intervention.

Li et al. (17) present an interesting paradox in the relationship between urbanization and myopia. While higher urbanization was associated with increased myopia incidence, it was also linked to slower myopia progression. This nuanced finding underscores the complex interplay between environmental factors and visual development, possibly reflecting the dual nature of urban environments in providing both risk factors (e.g., increased near-work activities) and protective elements (e.g., access to healthcare).

While the exact mechanisms through which green spaces protect against myopia are not fully elucidated, several hypotheses emerge from the reviewed studies. Increased time spent outdoors, often associated with exposure to green spaces, is a frequently mentioned as a key factor in Myopia prevention. Wang’s study (14) quantified this factor, showing that 2.7% of the protective effect of green spaces was mediated by increased outdoor time.

The primary limitation of this study lies in the challenge of distinguishing the effects of green spaces from other potential confounding factors, such as outdoor time, solar irradiation intensity, and physical activity. Leone et al. attempted to quantify outdoor time using questionnaires (11). However, these methods were found to lack accuracy and introduce significant bias. To more precisely isolate the effects of solar exposure from those of green spaces, it is recommended to use a more objective measurement method, such as conjunctival ultraviolet autofluorescence (CUVAF) (18).

Additionally, Yang et al. (13) found that lower ambient PM_1_ levels in greener areas mediated a significant portion of the protective effect, suggesting that improved air quality may serve as another pathway through which green spaces influence visual health.

It is important to note that most of these studies relied on non-cycloplegic refraction as the diagnosis method for myopia, which can potentially lead to an overestimation of myopia prevalence (19). Future studies utilizing cycloplegic refraction diagnosis could provide more accurate estimates of the relationship between green spaces and myopia.

The consistent protective effect of green spaces on myopia across various studies has significant implications for urban planning and public health policies. These findings support the benefits of integrating abundant, well-connected green spaces in the design of urban environments, particularly around schools and residential areas. Such interventions could serve as a cost-effective, population-level approach to myopia prevention.

Although NDVI utilizes reflectance from red and near-infrared light to quantify vegetation, it is important to distinguish this from studies investigating the effects of near-infrared light on myopia development (20). In this context, NDVI serves as a proxy for green space exposure, not as a measure of direct light exposure to the eye. Therefore, while both involve near-infrared wavelengths, they pertain to different mechanisms and should not be conflated.

In conclusion, the growing body of evidence strongly supports the role of green spaces in protecting against myopia and visual impairments in children and adolescents. These findings not only contribute to our understanding of environmental influences on eye health but also provide a strong rationale for integrating green space planning into public health strategies aimed at reducing the global burden of myopia.

## Future directions

While the current body of evidence regarding the protective effect of green spaces on Myopia prevalence is compelling, several research areas warrant further investigation. Longitudinal studies are necessary to establish causality and examine long-term effects of green space exposure on myopia development and progression, as well as regarding the specific characteristics of green spaces (e.g., type of vegetation, biodiversity) that are most beneficial for eye health. Finally, exploring and identifying the potential biological mechanisms underlying the effect of exposure to green space on eye development is crucial. This may involve investigating processes such as vitamin D synthesis, dopamine release, and other physiological processes.
